# Hospice Care Improves Patients’ Self-Decision Making and Reduces Aggressiveness of End-of-Life Care for Advanced Cancer Patients

**DOI:** 10.3390/ijerph192315593

**Published:** 2022-11-24

**Authors:** Chun-Li Wang, Chia-Yen Lin, Shun-Fa Yang

**Affiliations:** 1 Institute of Medicine, Chung Shan Medical University, Taichung 402, Taiwan; 2 Department of Family Medicine, Taichung Veterans General Hospital, Taichung 407, Taiwan; 3 Division of Urology, Department of Surgery, Taichung Veterans General Hospital, Taichung 407, Taiwan; 4 Department of Medical Research, Chung Shan Medical University Hospital, Taichung 402, Taiwan

**Keywords:** aggressiveness of cancer care at the end of life (ACCEoL), autonomy, hospice care, do-not-resuscitate (DNR), terminal cancer

## Abstract

The aim of the current study is to evaluate the different degrees of hospice care in improving patients’ autonomy in decision-making and reducing aggressiveness of cancer care in terminal-stage cancer patients, especially in reducing polypharmacy and excessive life-sustaining treatments. This was a retrospective cross-sectional study conducted in a single medical center in Taiwan. Patients with advanced cancer who died in 2010–2019 were included and classified into three subgroups: hospice ward admission, hospice shared care, and no hospice care involvement. In total, 8719 patients were enrolled, and 2097 (24.05%) admitted to hospice ward; 2107 (24.17%) received hospice shared care, and 4515 (51.78%) had no hospice care. Those admitted to hospice ward had significantly higher rates of having completed do-not-resuscitate order (100%, *p* < 0.001) and signed the do-not-resuscitate order by themselves (48.83%, *p* < 0.001), and they had lower aggressiveness of cancer care (2.2, *p* < 0.001) within the 28 days before death. Hospice ward admission, hospice shared care, and age > 79 years were negatively associated with aggressiveness of cancer care. In conclusion, our study showed that patients with end-of-life hospice care related to higher patient autonomy in decision-making and less excessively aggressive cancer care; the influence of care was more overt in patients approaching death. Further clinical efforts should be made to clarify the patient and the families’ satisfaction and perceptions of quality after hospice care involvement.

## 1. Introduction

Despite rapid advances in medical technology and drug treatment, cancer is the second leading cause of death worldwide, accounting for nearly 10 million deaths in 2020 [1]. Since 1982, cancer ranks first among causes of death in Taiwan, accounting for 28.6% of total deaths in 2019 [2]. Most cancer patients experience various clinical discomforts, including of a physical, psychological, and spiritual nature; examples are pain, shortness of beath, depression, and worthlessness [3,4,5]. Clinicians have expended considerable energy to address therapeutic possibilities, but “whole-person” care [6,7] problems are often neglected unintentionally, particularly among terminal-stage cancer patients.

Research on end-of-life care has recently been increasingly discussed, especially regarding aggressiveness of cancer care at the end of life [8,9,10,11,12,13,14,15]. Earle et al. [9,10,11,12] conducted one of the first studies, using indicators within the last 30 days of life to identify the trend of aggressiveness of cancer care; the indicators included overuse of chemotherapy, >1 emergency department visit, >1 hospital admission, intensive care unit admission, or underuse of hospice care. Martins-Branco et al. expanded the scope of aggressiveness of cancer care on the basis of a systematic review by Luta et al. [16] in 2020, categorizing 16 indicators into three dimensions [15]: healthcare resource use in the last 30 days of life, systemic antineoplastic treatments in the last 14 days of life, and advanced life support and/or life-prolonging procedures in the last 30 days of life. Although these indicators have been reviewed, other indispensable indicators of the intensity of cancer care during the terminal period, such as patients’ autonomy in decision-making and reducing polypharmacy and excessive life-sustaining treatments, have not been discussed.

Whether excessive aggressiveness of cancer care represents the poor quality of life was an open question [12]. Wright et al. [17] had used the McGill Quality of Life Index to assess the association between patients’ aggressive medical interventions and quality of life, which had showed negative correlation, whereas hospice care improved patients’ quality of life the longer they were enrolled. Towards the end of life, reducing aggressiveness of cancer care brings dignity and a sense of peace and safety to terminal cancer patients and their families. Hospice care helps intractable symptoms control, reduces invasive life-sustaining treatments, and improves patient and families awareness of disease prognosis [18,19], offering better end-of-life care to patients with advanced cancer [19,20,21,22,23,24]. Therefore, in recent decades, extensive attention has been paid to hospice care, with the aim of maintaining the quality of life of terminal-stage patients.

Hospice care in Taiwan has gradually advanced since the establishment of the first hospice ward in 1990. Ten years later, the Legislative Yuan in Taiwan had promulgated the Hospice Palliative Care Regulation, the law of safeguarding the terminal patients’ rights to choose “rejection of cardiopulmonary resuscitation” and life-sustaining treatments by signing a do-not-resuscitate order after two disease-related specialists confirm their terminal condition [25,26]. Taiwan is the first nation in Asia to formally and legally protect the rights to choose [27]. In these and the following years, hospice home care and hospice shared care became available in 1996 and 2011 [28], and their utilization increased markedly over the past 20 years [24]. Previous study showed the prevalence of hospice care utilization rose from 19.3% in 2008 to 41.9% in 2013 according to the databases of the National Death Registry [19].

This analysis evaluated the different degrees of hospice care involvement in reducing aggressiveness of cancer care in advanced and life-limiting cancer patients during the end-of-life period; special attention focused on whether hospice care improves patients’ autonomy in decision-making and reduces polypharmacy and excessive life-sustaining treatments.

## 2. Materials and Methods

### 2.1. Data Source and Study Patients

This was a retrospective cross-sectional study at Taichung Veterans General Hospital, a tertiary medical center in Taiwan. Data were anonymized by the Clinical Informatics Research and Development Centre (registered number: F20400). The study protocol was approved by the institutional review board (institutional review board number: CE20362A#1).

Patients who died of cancer between 2010 and 2019 were included. Patients who died before the age of 20 or died on the same day as hospice care was commenced were excluded. To investigate the correlation between varying degrees of hospice care and patient’s autonomy in decision-making and reduced aggressiveness of cancer care at the end of life, we divided the patients into three subgroups, regarding to the intensity of hospice care: hospice ward admission, hospice shared care, and no hospice care involvement.

**Figure 1:**
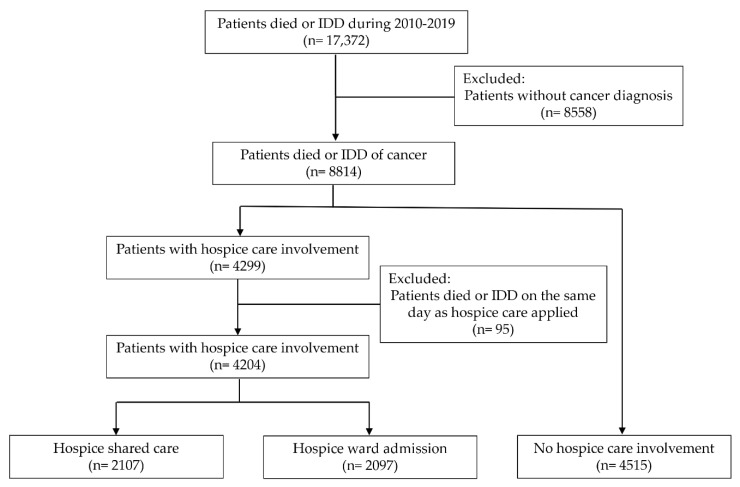
The flowchart of the study selection process. IDD, impending death discharge.

The hospice care team consists of a hospice specialist, nurse, counselling psychologist, religious teacher, and art therapist. The hospice care services in the hospice ward include (1) symptoms control: providing advice on appropriate drug management for common terminal symptoms. (2) Patient body care (edema massage, abdominal massage and wound dressing change, etc.), comfort care (positioning, bathing and shampooing in bed, pedicure care, skin care, oral care, passive exercise, and other treatment guidance such as diet guidance, rehabilitation guidance, etc.). (3) The care and referral of the psychosocial spiritual needs of patients and their families. (4) Assisting patients and their families in the disease cognition and decision-making of important treatment plan. (5) Facilitating communication between patients or their families. Hospice shared care was defined as under the care of the cancer specialists at a nonhospice ward and receiving hospice care by consulting the hospice care team [29]. Patients who had first received hospice shared care, followed by hospice ward admission, were classified as the hospice ward admission group. The study flowchart is presented in Figure 1.

### 2.2. Measures

We reviewed the patients’ demographic and clinical data, including gender, age at death, type of cancer diagnosis, place of death, status of do-not-resuscitate order, type of do-not-resuscitate order (signed by the patient or by the family proxy and legal surrogate), and comorbidities.

Primary outcomes were the patient’ s right of self-decision making, defining as the percentage of completed do-not-resuscitate orders and the percentage of do-not-resuscitate orders signed by the patient, and the occurrence of 16 indicators of aggressiveness of cancer care in the three subgroups. Based on the 16 indicators proposed by Martins-Branco et al. [15], we designed our 16 indicators in three dimensions according to the medical culture in Taiwan. The three dimensions and the indicators were (1) healthcare resource use: >1 hospitalization, >14 days of hospitalization, ≥1 intensive care unit admission, and ≥1 emergency department visit; (2) aggressive medication administration: systemic antineoplastic drug administration and excessive medication administration; and (3) life-sustaining treatments: cardiopulmonary resuscitation, endotracheal tube insertion with mechanical ventilation, vasopressor support, tracheostomy, hemodialysis, nasogastric tube placement, central vascular catheter implantation, excessive parenteral infusion of nutrition, imbalance of input and output fluid, and surgical intervention.

We defined excessive medication administration, excessive parenteral infusion of nutrition, and imbalance of input and output fluid as more than their median in all included populations. The definite number of these three items was also calculated. All indicators were analyzed within 28 days of death, except for excessive parenteral infusion of nutrition and imbalance of input and output fluid, which were analyzed in the last 7 days of life.

### 2.3. Statistical Analysis

Owing to the non-normal distribution analyzing by Kolmogorov–Smirnov test firstly, we used the Kruskal–Wallis test or chi-square test to compare the characteristics of patients in the three subgroups with different statuses and types of hospice care involvement. Categorical data were expressed as numbers and percentages, and continuous variables were expressed as means ± standard deviations. We then calculated the number of occurrences of the total and each indicator in the last 7 or 28 days of life, comparing the differences between the three subgroups. A *p* value of <0.05 was considered statistically significant. Moreover, we used generalized estimating equations to calculate the rate and quantity estimation on the 28th, 14th, 7th, 5th, and 3rd day before death and the day of death.

We compared the patient with occurrence of total indicators in the first quarter to the remaining three quarters and tried to analyze the impact of different degrees of hospice involvement. We used the Mann–Whitney U test, chi-square test, and Fisher’s exact test to examine the associations between all categorical variables and the group with higher aggressiveness of cancer care. To determine the independent variables of high aggressiveness of cancer care, we used univariate and multivariate logistic regression models. All statistical analyses were performed using SPSS (IBM SPSS version 22.0; International Business Machines Corp., New York, NY, USA).

## 3. Results

### 3.1. Patient Characteristics by Status and Type of Hospice Care

A total of 8719 patients were enrolled (male patients: 66%; mean age: 65.4 years). The average hospitalization duration before death was 19.56 days (SD: ±20.11). The most common cancers were those of the lung and liver, followed by hematologic malignancies. More than half of the patients died in the hospital (Table 1).

By status and type of hospice care, all patients were divided into three subgroups (Table 1); 2097 (24.05%) were admitted to a hospice ward, 2107 (24.17%) received hospice shared care, and 4515 (51.78%) received no hospice care. Regarding gender in each subgroup, the hospice ward admission group had the highest female proportion (*p* = 0.002). Regarding cancer diagnosis, the three most common cancers in the groups of hospice shared care and no hospice care were lung (30.09% and 29.28%, respectively), liver (18.51% and 19.58%, respectively), and hematologic malignancies (9.63% and 14.62%, respectively), which were different from those in the hospice ward admission group (lung (18.50%), liver (18.45%), and head and neck cancers (12.88%)). In the hospice ward admission group, hematologic malignancies ranked eleventh (4.01%). This group had a significantly higher incidence of death in the hospital (hospice ward, 80.83%; hospice shared care, 50.17%; no hospice care, 45.16%; *p* < 0.001). Regarding comorbidities, patients with chronic kidney disease, chronic obstructive pulmonary disease, and liver cirrhosis mostly accounted for the majority in the group without hospice care (Table 1).

### 3.2. Primary Outcome

**Figure 2:**
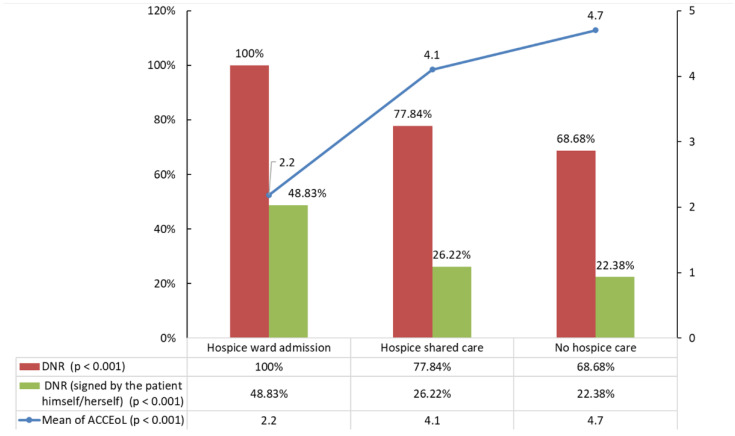
The percentage of completed DNR order and DNR order signed by the patient and the mean ACCEoL numbers by the status and type of hospice care. DNR, do-not-resuscitate; ACCEoL, aggressiveness of cancer care at the end of life.

The average occurrence of all indicators of aggressiveness of cancer care in all patients was 3.95 (±2.29), but it was significantly lower in the hospice ward admission group (2.2, *p* < 0.001). In total, 6838 (78.43%) patients had signed a do-not-resuscitate order. The percentage of those signing a do-not-resuscitate order was significantly higher in the hospice care groups (100%, 77.84%, vs. 68.68%, *p* < 0.001). In all included populations, more than half the patients in each group had their do-not-resuscitate order signed by someone other than themselves. However, the difference between do-not-resuscitate orders signed by the patients themselves (48.83%, 26.22%, vs. 22.38%, *p* < 0.001) and by family or a surrogate in the hospice ward admission group was only approximately 3% (Figure 2).

### 3.3. Indicators of Aggressiveness of Cancer Care at the End of Life

**Figure 3:**
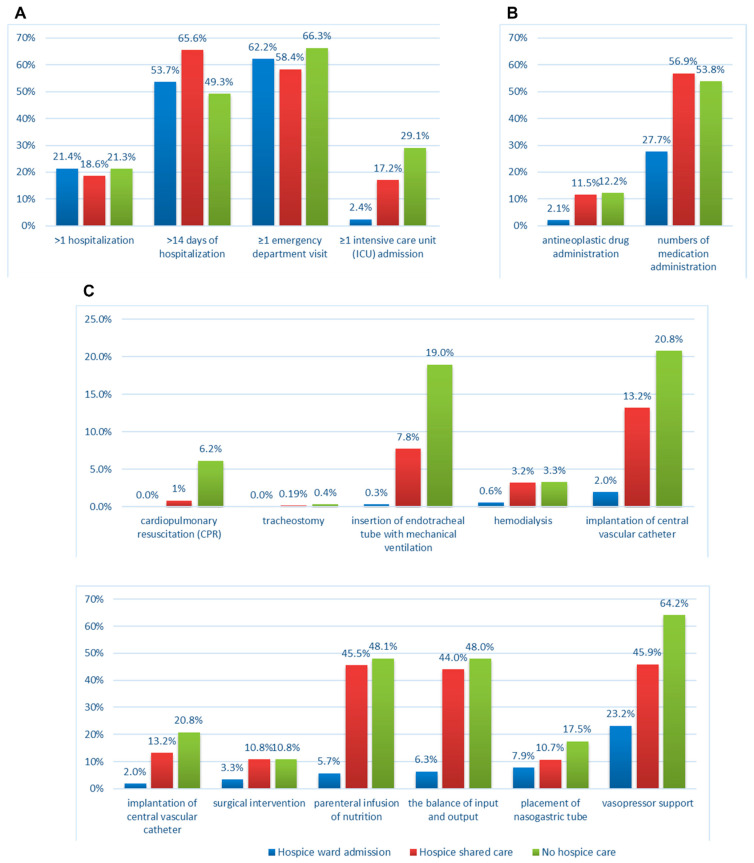
The prevalence of individual ACCEoL indicators by the status and type of hospice care. (**A**) Healthcare resources use, (**B**) aggressive medication administration, (**C**) life-sustaining treatments.

Within the 28 days before death, the prevalence of indicators among the three subgroups significantly differed (Table 2; Figure 3). The hospice ward admission and hospice shared care groups exhibited a significant reduction in most indicators, namely, ≥1 intensive care unit admission and ≥1 emergency department visits in the dimension of healthcare resource use, systemic antineoplastic drug administration in the dimension of aggressive medication administration, and all indicators in the dimension of life-sustaining treatments, except for undergoing tracheostomy (*p* < 0.001).

Among all the indicators, >1 hospitalization occurred most often in the hospice ward admission group (21.41%, *p* = 0.025), and the hospice shared care group had the highest prevalence of >14 days of hospitalization (65.59%, *p* < 0.001) and excessive medication administration (56.58%, *p* < 0.001).

**Figure 4:**
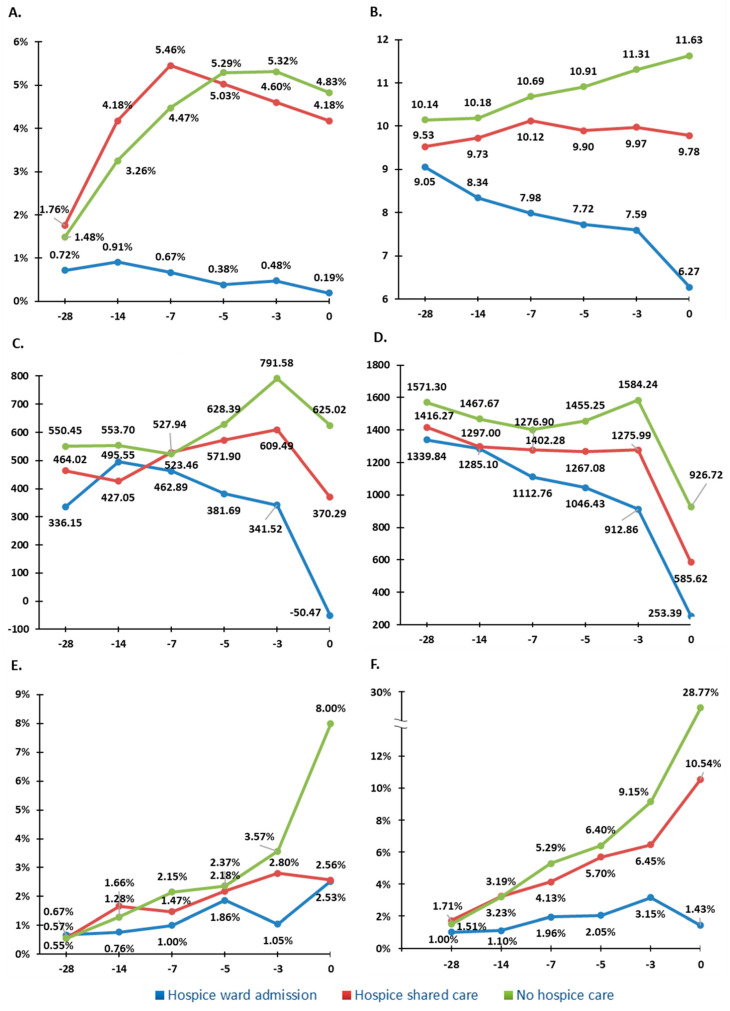
The trend of rate and the quantity of the indicators on the 28th, 14th, 7th, 5th, and 3rd day before death and the day of death. The *X*-axis represents the days before death. The *Y*-axis represents the percentage of antineoplastic drug administration in (**A**), the numbers of medication in (**B**), the imbalance amount (mL) of input and output fluid, in (**C**), the amount (mL) of parental infusion fluid in (**D**), the percentage of nasogastric tube insertion (**E**), and the percentage of vasopressor support in (**F**).

In addition to the lower prevalence of indicators, the trend of rate and quantity of the indicators including aggressive medication administration and life-sustaining treatments on the 28th, 14th, 7th, 5th, and 3rd day before death and the day of death was significantly lower in the hospice shared care and hospice ward admission groups (Figure 4).

### 3.4. Factors Associated with Aggressiveness of Cancer Care

The included populations were divided into two subgroups according to the number of occurrences of indicators, with >5 as the cut-point, the 25th percentile of indicators occurrence distribution in our cohort. Table 3 presents the clinical parameters and the type and status of hospice care involvement in the two subgroups. In total, 6651 patients had ≤5 items of aggressiveness of cancer care (group 1) and 2068 patients had >5 items (group 2). The proportion of patients admitted to the hospice ward (31.12% vs. 1.31%; *p* < 0.001) and older patients (age: >79 years) were considerably higher in group 1. By contrast, the proportion of patients without hospice care involvement, male patients, and younger patients (age: 20–39 years) was significantly higher in group 2. The proportion of patients who died in the hospital (57.87% vs. 45.55%; *p* < 0.001), had signed a do-not-resuscitate order (79.54% vs. 74.85%; *p* < 0.001), and signed their own do-not-resuscitate order (35.92% vs. 16.02%; *p* < 0.001) was higher in group 1. The proportion of patients who had their do-not-resuscitate orders signed by family or a surrogate was higher in group 2 (83.98% vs. 64.08%; *p* < 0.001).

In multivariate analysis, hospice shared care (OR 0.44; 95% CI 0.37–0.51, *p* < 0.001), hospice ward admission (OR 0.02; 95% CI 0.01–0.03, *p* < 0.001), and older age (>79 years; OR 0.62; 95% CI 0.43–0.89, *p* = 0.009) were negatively associated with aggressiveness of cancer care at the end of life. Male patients (OR 1.27; 95% CI 1.09–1.47, *p* = 0.002) and patients who did not sign their do-not-resuscitate orders (OR 1.23; 95% CI 1.09–1.39, *p* = 0.001) or those whose do-not-resuscitate orders were signed by family or a surrogate (OR 1.94; 95% CI 1.64–2.29, *p* < 0.001) tended to have high aggressiveness of cancer care (Table 4).

## 4. Discussion

Using modified established indicators, we examined the impact of different degrees of hospice care in reducing aggressiveness of cancer care. In our study, hospice care, especially hospice ward admission, improved patient’s medical autonomy in decision-making and led to a reduction in the aggressiveness of care, including reducing emergency department visits, intensive care unit admissions, antineoplastic drug administration, and most of the life-sustaining treatments among patients with advanced cancer at the end of life. Researches took indicators of aggressive care as quality indicators of end-of-life care [13,16,24,30,31]. Earle et al. [9] stated continuation of antineoplastic agent and intensive admission or emergency room visiting near death as two of the major concepts of poor-quality of end-of-life care. Previous studies showed hospice care decreased aggressive end-of-life care [14,18,19,24], improved the quality of life [32,33,34], upgraded the end-of-life satisfaction [35,36], and saved the medical costs [37,38,39,40]. Our study more comprehensively evaluated treatment programs conducted under hospice care that may benefit terminal patients.

Besides physical discomforts, terminal cancer patients face the loss of autonomy and dignity, which severely destroys the patient’s emotions and spirituality. Our study demonstrated a statistically significant proportion of hospice care group patients completed a do-not-resuscitate order, as well as the percentage of do-not-resuscitate orders signed by the patient. The definition of patient autonomy at the end of life had been reviewed [41] and two core structural values of autonomy were identified: (1) being normal and (2) taking charge. Self decision-making is one of the items in the second part, and also important to the patient and the families [42]. The orders were more often signed by family or a surrogate in all populations and in the three subgroups. Similar to other East Asian countries [43], cultural factors may contribute to the results that the families were explained and discussed the disease problems first while some physicians value the families’ opinion more than the patient. Nevertheless, hospice care involvement, especially hospice ward admission, reduced the regret of not being able to decide for yourself.

Among the four indicators of healthcare resource use, hospice ward admission ranked first in >1 hospitalization and second in >14 days of hospitalization and ≥1 emergency department visit. Moreover, 80% of patients from the hospice ward admission group died in the hospital instead of at home, which has been regarded as a preferred place of death and constitutive of a good death [44,45,46,47,48]. One possible reason for this preference is the rapid deterioration and refractory symptoms of patients. Most patients with advanced cancer experience symptoms throughout the disease course, which often worsen as they approach death [49,50,51,52]. Studies have reported that patient-centered home care could reduce unnecessary emergency visits and hospitalizations [53,54]. The high frequency and length of hospitalization and emergency room visits may reflect the need for improving community support with respect to physical symptom relief, psychological emotion support, and social acceptance.

Our study reported a decreasing number of medications in the hospice ward admission group as death approached, which was the opposite of the trend in the no hospice care intervention group. Owing to old age, a progressive disease course, and multiple comorbidities, patients with advanced cancer are often prescribed excessive medications [55,56]. The mean number of drugs prescribed for terminal patients is 15.7 (range 1–100) in the United States [57] and 8 (range 0–17) in Northern Ireland [58]. The consequences of polypharmacy in terminal patients are increased risk of drug interactions, adverse effects, and medical costs [55,59]. Several classes of drugs can be discontinued in terminal patients [60,61], especially those used for primary or secondary prevention. The OncPal deprescribing guideline is useful for assisting physicians in de-escalating inappropriate medications for patients with advanced cancer who have a limited life expectancy [62].

As indicators of life-sustaining treatments, we first discussed artificial nutrition, including nasogastric tube placement, excessive parenteral infusion of nutrition, and the imbalance of input and output fluids. Most terminal-stage patients experience dry mouth, cachexia, and weakness, and many of the patients and their family members themselves wish to have artificial nutrition and hydration [63,64,65]. Studies have discovered that artificial nutrition and hydration does not influence survival [64,66] or provide comfort to terminally ill patients [67]. Patients with advanced cancer in the hospice ward group received a gradually decreased amount of parenteral infusion fluid in the last 28 days of life, with balance and less difference between input and output amount. By contrast, patients in the other two groups had increasingly imbalanced amount of fluid when death approached. The increasing percentage of nasogastric tube placement in all three groups may have resulted from the preparation for drug administration under home care.

In the multivariable analysis of our study, the hospice ward admission group showed the lowest likelihood of receiving inappropriately aggressive cancer care. Admission to a hospice ward with specialists is the most helpful approach to reduce futile medical interventions. This indicates that access to hospice care must be broadened. The do-not-resuscitate orders signed by patients themselves were associated with lower aggressiveness of cancer care. This means that when patients were more aware of their own disease conditions, they favored a good death; preferences for the dying process, including the death scene (how, who, where, and when), and preparations for death were the most recognized core themes of a good death [68]. While the patients were made comfortable, hospice care also lowered medical costs [69] without compromising survival time [22,70,71,72].

Our finding of the different gender distribution in three groups showed a higher preference for female patients enrolling hospice care, which was consistent with the prior research [73,74,75]. The gender discrepancy may relate to the social and self-expectancy toward the role socialization; male tends to be more aggressive, fearless, and indefatigable when facing the uncurable cancer [75]. Our study showed the patients with additional chronic illness were less likely to be referred to the hospice care, this may result from the more complex medical need from the subspecialist [76]. However, we could still clarify whether the physician’s preference, medical routine, or the not popularized hospice concept in other departments contributes to the heterogeneity in the comorbidity in three groups.

As part of the ongoing advocacy efforts for increased awareness of patient autonomy in recent decades, the Patient Right to Autonomy Act, the first law fully protecting patients’ autonomy, was passed in 2019. It emphasizes patient autonomy and allows patients in specific clinical conditions to decide their program of medical care including “life-sustaining treatments” and “artificial nutrition” in advance by attending advance care planning and signing an advance decision. In future, we look forward to more patient autonomy in medical decision-making.

This study has several strengths. Firstly, our study contributes to the literature by examining the influence of hospice care in patient autonomy in decision-making and aggressive cancer care among deceased patients with terminal cancer. Secondly, we are the first study exploring the different intensity of hospice care on the issue of end-of-life cancer care, which shows a direct association to the results. Thirdly, we have a long recruitment period with large sample size, conducting the clinical real-world, detailed, and comprehensive result outcome, comparing to the national health insurance database analysis study.

Our study has some limitations. It was a retrospective single-center study, which restricts the generalization of our results. Moreover, it was database-based research; therefore, we could not determine whether the decisions on end-of-life care arose from patients’ preferences, physicians’ routine, or medical resource availability. Among the indicators, the indications of surgery were not identified, which may be a palliative surgery, such as a palliative orthopedic surgery for skeletal metastases. Finally, further study is needed to estimate the effect of aggressive care on outcomes such as overall survival, patients’ perceptions of quality of care and their satisfaction with care.

As we could discover in this retrospective study, though Taiwan has been an advanced nation in the Asia–Pacific region in the issues of hospice care, there are still over half of the patients in terminal cancer stage who did not receive hospice care. Our study illustrated the findings that with the increased participation in hospice care, there could be less aggressive cancer care, and, thus, there may be the opportunity to create better quality of life of terminal cancer patients. We still have a long way to go; henceforth, we need to strive for an escalation in promoting the concept of hospice care, both to the clinicians and the public. Our study might help physicians in clinical treating cancer patients; future randomized control trail or meta-analysis are needed to draw firm evidence about the overall outcome and quality of life.

## 5. Conclusions

In conclusion, among adults with advanced cancer and a limited lifespan, hospice care led to improvement of patients’ medical autonomy in decision-making and reduction in aggressiveness of care. Our study demonstrated that hospice care, especially comprehensive care in a hospice ward, reduced polypharmacy, antineoplastic drug administration, and excessive life-sustaining treatments in the terminal stage of life. Its influence was more significant in patients approaching death. In the meantime, patient and the families’ satisfaction and perceptions of quality after hospice care involvement could be investigated in the next study. As hospice care offers a dignified death to patients with a terminal illness, future efforts are required to enhance the continuity and coordination of hospice care in the community.

## Figures and Tables

**Table 1:** Patient characteristics by the status and type of hospice care.

	Total (n = 8719)		Hospice Shared Care (n = 2107)		Hospice Ward Admission (n = 2097)		No Hospice Care Involvement (n = 4515)		*p* Value
Age	65.42	±14.21	64.46	±13.82	65.64	±14.48	65.76	±14.24	0.001 **
Gender									0.002 **
Female	2952	(33.86%)	728	(34.55%)	767	(36.58%)	1457	(32.27%)	
Male	5767	(66.14%)	1379	(65.45%)	1330	(63.42%)	3058	(67.73%)	
Length of last admission stay	19.56	±20.11	23.85	±21.60	18.97	±19.50	17.82	±19.37	<0.001 **
Cancer diagnosis									<0.001 **
Lung	2344	(26.88%)	634	(30.09%)	388	(18.50%)	1322	(29.28%)	
Liver	1661	(19.05%)	390	(18.51%)	387	(18.45%)	884	(19.58%)	
Hematologic	947	(10.86%)	203	(9.63%)	84	(4.01%)	660	(14.62%)	
Colorectal	676	(7.75%)	177	(8.40%)	212	(10.11%)	287	(6.36%)	
Head and neck	721	(8.27%)	153	(7.26%)	270	(12.88%)	298	(6.60%)	
Esophageal	399	(4.58%)	92	(4.37%)	117	(5.58%)	190	(4.21%)	
Gastric	350	(4.01%)	81	(3.84%)	114	(5.44%)	155	(3.43%)	
GYN cancer	259	(2.97%)	55	(2.61%)	88	(4.20%)	116	(2.57%)	
Breast	203	(2.33%)	40	(1.90%)	85	(4.05%)	78	(1.73%)	
Prostate	184	(2.11%)	31	(1.47%)	60	(2.86%)	93	(2.06%)	
Bladder	175	(2.01%)	26	(1.23%)	51	(2.43%)	98	(2.17%)	
Pancreases	406	(4.66%)	117	(5.55%)	138	(6.58%)	151	(3.34%)	
Other	394	(4.52%)	108	(5.13%)	103	(4.91%)	183	(4.05%)	
Comorbidity									
Ischemic heart diseases	1298	(14.89%)	296	(14.05%)	304	(14.50%)	698	(15.46%)	0.274
Cerebral infarction	313	(3.59%)	95	(4.51%)	76	(3.62%)	142	(3.15%)	0.021 *
Hypertension	3907	(44.81%)	907	(43.05%)	958	(45.68%)	2042	(45.23%)	0.164
T2DM	2340	(26.84%)	559	(26.53%)	530	(25.27%)	1251	(27.71%)	0.108
CKD	2765	(31.71%)	829	(39.35%)	618	(29.47%)	1318	(29.19%)	<0.001 **
COPD	1791	(20.54%)	403	(19.13%)	390	(18.60%)	998	(22.10%)	0.001 **
Liver cirrhosis	1717	(19.69%)	405	(19.22%)	367	(17.50%)	945	(20.93%)	0.004 **
Place of death									<0.001 **
Home	3928	(45.05%)	1050	(49.83%)	402	(19.17%)	2476	(54.84%)	
Hospital	4791	(54.95%)	1057	(50.17%)	1695	(80.83%)	2039	(45.16%)	

Values were expressed as numbers and percentages (or mean and SD). CKD, chronic kidney disease; COPD, chronic obstructive pulmonary disease; DM, diabetes mellitus; DNR, do-not-resuscitate; GYN, gynecological. * *p* < 0.05, ** *p* < 0.01.

**Table 2:** The prevalence of individual ACCEoL indicators by the status and type of hospice care.

	Hospice Shared Care (n = 2107)		Hospice Ward Admission (n = 2097)		No Hospice Care Involvement (n = 4515)		*p* Value
Healthcare resources use							
>1 hospitalization	391	(18.56%)	449	(21%)	960	(21.26%)	0.025 *
>14 days of hospitalization	1382	(65.59%)	1126	(54%)	2227	(49.32%)	<0.001 **
≥1 emergency department visit	1230	(58.38%)	1305	(62%)	2994	(66.31%)	<0.001 **
≥1 intensive care unit admission	362	(17.18%)	51	(2%)	1313	(29.08%)	<0.001 **
Aggressive medication administration							
antineoplastic drug administration	243	(11.53%)	44	(2%)	553	(12.25%)	<0.001 **
excessive numbers of medication administration	1198	(56.86%)	580	(28%)	2430	(53.82%)	<0.001 **
Life-sustaining treatments							
cardiopulmonary resuscitation	18	(0.85%)	0	(0%)	278	(6.16%)	<0.001 **
insertion of endotracheal tube with mechanical ventilation	164	(7.78%)	7	(0%)	856	(18.96%)	<0.001 **
vasopressor support	967	(45.89%)	486	(23%)	2899	(64.21%)	<0.001 **
tracheostomy	4	(0.19%)	1	(0%)	16	(0.35%)	0.052
placement of nasogastric tube	226	(10.73%)	165	(8%)	791	(17.52%)	<0.001 **
implantation of central vascular catheter	278	(13.19%)	41	(2%)	939	(20.80%)	<0.001 **
excessive parenteral infusion of nutrition	959	(45.51%)	120	(6%)	2171	(48.08%)	<0.001 **
the imbalance of input and output fluid	927	(44.00%)	133	(6%)	2168	(48.02%)	<0.001 **
surgical intervention	227	(10.77%)	70	(3%)	489	(10.83%)	<0.001 **
hemodialysis	68	(3.23%)	12	(1%)	149	(3.30%)	<0.001 **

* *p* < 0.05, ** *p* < 0.01.

**Table 3:** Clinical characteristics and status and type of hospice care of participants by the occurrences of ACCEoL.

	ACCEoL ≤ 5 (n = 6651)		ACCEoL > 5 (n = 2068)		*p* Value
Status and type of hospice care					<0.001 **
hospice shared care	1622	(24.39%)	485	(23.45%)	
hospice ward admission	2070	(31.12%)	27	(1.31%)	
no hospice care	2959	(44.49%)	1556	(75.24%)	
Age	65.71	±14.18	64.47	±14.26	0.003 **
Age					<0.001 **
20–39	236	(3.55%)	111	(5.37%)	
40–59	2120	(31.87%)	653	(31.58%)	
60–79	3078	(46.28%)	980	(47.39%)	
>79	1217	(18.30%)	324	(15.67%)	
Gender					<0.001 **
Female	2339	(35.17%)	613	(29.64%)	
Male	4312	(64.83%)	1455	(70.36%)	
Cancer diagnosis					<0.001 **
Lung	1878	(28.24%)	466	(22.53%)	
Liver	1323	(19.89%)	338	(16.34%)	
Hematologic	537	(8.07%)	410	(19.83%)	
Colorectal	490	(7.37%)	186	(8.99%)	
Head and neck	576	(8.66%)	145	(7.01%)	
Esophageal	284	(4.27%)	115	(5.56%)	
Gastric	290	(4.36%)	60	(2.90%)	
GYN cancer	208	(3.13%)	51	(2.47%)	
Breast	177	(2.66%)	26	(1.26%)	
Prostate	149	(2.24%)	35	(1.69%)	
Bladder	113	(1.70%)	62	(3.00%)	
Pancreases	346	(5.20%)	60	(2.90%)	
Other	280	(4.21%)	114	(5.51%)	
Death in the hospital	3849	(57.87%)	942	(45.55%)	<0.001 **
Signed DNR order	5290	(79.54%)	1548	(74.85%)	<0.001 **
Type of DNR order					<0.001 **
Signed by the patient	1900	(35.92%)	248	(16.02%)	
Signed by the family or surrogate	3390	(64.08%)	1300	(83.98%)	

GYN, gynecological; DNR, do-not-resuscitate. ** *p* < 0.01.

**Table 4:** Univariate and multivariate logistic regression analysis: independent variables assessed high ACCEoL occurrence (ACCEoL > 5).

	Simple Model			Multiple Model		
	OR	95% CI	*p* Value	OR	95% CI	*p* Value
Status and type of hospice care						
Hospice shared care	0.57	(0.51–0.64)	<0.001 **	0.44	(0.37–0.51)	<0.001 **
Hospice ward admission	0.02	(0.02–0.04)	<0.001 **	0.02	(0.01–0.03)	<0.001 **
No hospice care	ref.			ref.		
Gender						
Female	ref.			ref.		
Male	1.29	(1.16–1.43)	<0.001 **	1.27	(1.09–1.47)	0.002 **
Age						
18–39	ref.			ref.		
40–59	0.65	(0.51–0.83)	0.001 **	0.72	(0.52–1.01)	0.056
60–79	0.68	(0.53–0.86)	0.001 **	0.75	(0.54–1.05)	0.094
>79	0.57	(0.44–0.73)	<0.001 **	0.62	(0.43–0.89)	0.009 **
*p* for trend	0.001 **			0.005 **		
Cancer diagnosis						
Lung	ref.			ref.		
Liver	1.03	(0.88–1.20)	0.715	1.11	(0.91–1.35)	0.307
Hematologic	3.08	(2.61–3.62)	<0.001 **	2.37	(1.93–2.92)	<0.001 **
Colorectal	1.53	(1.26–1.86)	<0.001 **	2.31	(1.79–2.99)	<0.001 **
Head and neck	1.01	(0.82–1.25)	0.892	1.71	(1.30–2.26)	<0.001 **
Esophageal	1.63	(1.28–2.07)	<0.001 **	2.42	(1.74–3.35)	<0.001 **
Gastric	0.83	(0.62–1.12)	0.229	0.87	(0.59–1.29)	0.502
GYN cancer	0.99	(0.72–1.36)	0.942	1.59	(1.03–2.47)	0.038 *
Breast	0.59	(0.39–0.90)	0.015 *	0.89	(0.50–1.55)	0.670
Prostate	0.95	(0.65–1.39)	0.778	1.00	(0.59–1.71)	0.997
Bladder	2.21	(1.60–3.06)	<0.001 **	2.62	(1.63–4.21)	<0.001 **
Pancreases	0.70	(0.52–0.94)	0.016 *	0.90	(0.63–1.28)	0.559
Other	1.64	(1.29–2.09)	<0.001 **	1.65	(1.19–2.27)	0.002 **
Comorbidity						
Ischemic heart diseases	1.17	(1.03–1.34)	0.019*	1.25	(1.04–1.51)	0.020 *
Cerebral infarction	0.84	(0.63–1.11)	0.212			
Hypertension	0.94	(0.85–1.04)	0.212			
T2DM	1.03	(0.92–1.15)	0.650			
CKD	1.06	(0.96–1.18)	0.252			
COPD	0.95	(0.84–1.08)	0.462			
Liver cirrhosis	0.96	(0.85–1.09)	0.517			
Place of death						
Home	ref.			ref.		
Hospital	0.61	(0.55–0.67)	<0.001 **	0.92	(0.80–1.05)	0.197
Signed DNR order						
No	1.31	(1.16–1.47)	<0.001 **	1.23	(1.09–1.39)	0.001 **
Yes	ref.			ref.		
Type of DNR order						
Signed by the patient	ref.			ref.		
Signed by the family or surrogate	2.94	(2.54–3.40)	<0.001 **	1.94	(1.64–2.29)	<0.001 **

OR, odds ratio; CI, confidence interval; CKD, chronic kidney disease; COPD, chronic obstructive pulmonary disease; DM, diabetes mellitus; DNR, do-not-resuscitate; GYN, gynecological. * *p* < 0.05, ** *p* < 0.01.

## Data Availability

The datasets generated for this study are available on request to the corresponding authors.
